# Efficacy of Indigenous Strains of Entomopathogenic Nematodes in Controlling the Eggplant Fruit and Shoot Borer *Leucinodes orbonalis*

**DOI:** 10.3390/insects16030272

**Published:** 2025-03-05

**Authors:** Salma Javed, Sajjad Ali, Connor J. Goldy, Bushra Nawab, Anil Baniya, Adler R. Dillman

**Affiliations:** 1 National Nematological Research Centre, University of Karachi, Karachi 75270, Pakistan; sajjadali9487@gmail.com; 2 Department of Nematology, University of California, Riverside, CA 92521, USA; cgold019@ucr.edu (C.J.G.); abaniya@ucr.edu (A.B.); adlerd@ucr.edu (A.R.D.); 3 Pakistan Council of Scientific and Industrial Research Council, Karachi 75280, Pakistan; bushra_nawab2003@yahoo.com

**Keywords:** *Steinernema pakistanense*, *Heterorhabditis indica*, biological control, *Leucinodes orbonalis*, PCR sequencing, field trials, eggplant

## Abstract

The growing reliance on chemical pesticides in agriculture has raised significant concerns about their environmental and health impacts. This study investigates the potential of Indigenous entomopathogenic nematodes (EPNs), specifically *Steinernema pakistanense* and *Heterorhabditis indica*, as biological control agents for *Leucinodes orbonalis*, the brinjal fruit and shoot borer, a major pest in Karachi, Sindh, Pakistan. A comprehensive soil sampling was carried out across 30 locations. Molecular identification via PCR and sequencing confirmed the taxonomic classification of the isolated strains, showing high genetic similarity to known EPN species. The reproductive potential of the isolated strains varied, with Pak.S.SA.22 exhibiting the highest fecundity, averaging 91,944 offspring per larva, indicating strong biocontrol potential. Laboratory bioassays at different concentrations showed that Pak.S.SA.22 achieved 100% mortality of *L. orbonalis* at a concentration of 200 IJs/mL, confirming its effectiveness. Field trials further validated these findings, demonstrating up to 90% pest control efficacy over multiple application intervals, highlighting the practical applicability of this strain in field conditions. These results advocate for the inclusion of indigenous EPNs in integrated pest management (IPM) programs as a sustainable, environmentally friendly alternative to chemical pesticides, contributing to the promotion of sustainable agricultural practices.

## Simple Summary

Eggplant (*Solanum melongena* L.) is an important crop in many tropical and subtropical regions of South Asia, valued for its economic significance and rich nutritional content, including vitamins, minerals, and antioxidants. It plays a crucial role in food security and rural livelihoods. However, one of the most destructive pests affecting eggplant productivity is the brinjal fruit and shoot borer (*Leucinodes orbonalis* Guenée), which causes severe crop losses. This pest has a broad host range and is widespread across various regions in Asia and Africa, leading to substantial economic damage to different crops. Managing this pest is particularly challenging as it burrows into plant tissues, making conventional control methods ineffective. As a result, large quantities of chemical insecticides are required, posing serious risks to human health and the environment while failing to provide long-term pest control. In this study, soil samples were collected from various locations in Pakistan to identify native species of entomopathogenic nematodes (EPNs). These native EPNs are well adapted to local soil conditions and have the potential to serve as a sustainable and long-term solution for managing this pest. During our survey, we isolated eight different EPN strains from two genera, *Steinernema* spp. and *Heterorhabditis* spp., from diverse vegetation types and evaluated their effectiveness in controlling the brinjal fruit and shoot borer. Additionally, field trials were conducted to assess whether the efficacy observed in laboratory experiments translated to real-world conditions. Our findings demonstrated that native EPNs exhibited high reproduction rates and were highly effective in controlling the brinjal fruit and shoot borer under both laboratory and field conditions.

## 1. Introduction

Eggplant (*Solanum melongena* L.), also known as brinjal or aubergine, holds significant importance in the agricultural economy of many tropical and subtropical countries. It is a rich source of vitamins, minerals, and antioxidants, contributing to food security and rural livelihoods [1]. However, the productivity of brinjal is severely threatened by the brinjal fruit and shoot borer (*Leucinodes orbonalis* Guenée), a notorious pest responsible for devastating crop losses. This pest primarily damages the shoots and fruits of the plant, making it unmarketable and causing a substantial decline in both yield and quality [2]. This pest is found extensively in Malaysia, Myanmar, Sri Lanka, India, Pakistan, Germany, and East Africa [3]. The larvae of *L. orbonalis* bore into the tender shoots and fruits, creating galleries that impede plant growth and fruit development. The pest’s ability to hide within plant tissues and its rapid lifecycle complicate control measures [4].

Conventional pest management for *L. orbonalis* relies heavily on the application of chemical insecticides. While these insecticides may provide temporary relief, their excessive use has led to several negative consequences, including the development of insecticide resistance, outbreaks of secondary pests, and harmful impacts on non-target organisms, including beneficial insects and natural enemies of pests [5]. Additionally, pesticide residues on brinjal pose serious health risks to consumers and contribute to environmental pollution, necessitating the search for more sustainable pest management solutions [6].

Entomopathogenic nematodes (EPNs) are microscopic roundworms, primarily from the families Steinernematidae and Heterorhabditidae, that have proven effective in controlling a wide range of insect pests. These nematodes, together with their symbiotic bacteria, kill their insect hosts by releasing toxins inside the host’s body, leading to septicemia and death within 48 h [7]. EPNs have the advantages of being eco-friendly, highly specific to insect pests, and safe for humans, animals, and beneficial insects. Moreover, the application of indigenous EPN strains is thought to offer further benefits, as these strains are well-adapted to local environmental conditions, enhancing their survival and efficacy in the field. It is suggested that the use of Indigenous strains not only reduces the risk of introducing exotic species into the local ecosystem but also increases the likelihood of successful pest control in the target region [8].

While EPNs have demonstrated significant efficacy in laboratory conditions, their performance in field settings is subject to various factors, including environmental conditions, soil type, and pest population dynamics. Hence, there is a growing need to evaluate the effectiveness of locally sourced EPN strains in real-world agricultural settings [9].

This study aims to bridge this gap by evaluating the efficacy of Indigenous strains of EPNs in controlling *L. orbonalis* under laboratory and microplot conditions. By assessing these EPNs’ pathogenicity, reproductive potential, and adaptability, the research seeks to provide an eco-friendly solution for managing *L. orbonalis* in Pakistan’s agroecosystems. The findings will contribute to the development of integrated pest management (IPM) strategies, reducing reliance on chemical pesticides and promoting sustainable agriculture.

## 2. Materials and Methods

### 2.1. Field Survey

**Figure 1:**
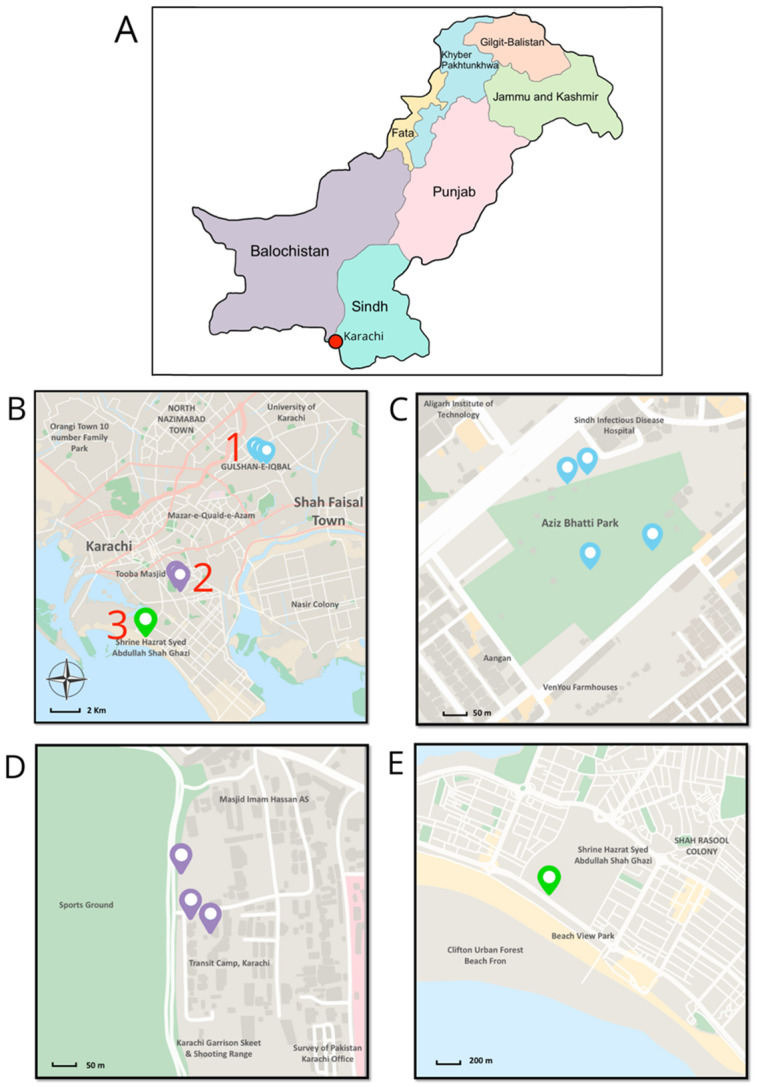
Nematode sampling locations in Karachi, Pakistan. (**A**) Map of Pakistan with Karachi marked by a red dot, indicating the sampling region. (**B**) Enlarged map of Karachi with three sampling sites marked. (**C**) Detailed nematode sampling location within site 1, Aziz Bhatti Park. (**D**) The detailed nematode sampling location is Site 2, Family Park Sabir SRE. (**E**) Detailed nematode sampling location in Site 3, Bagh Ibn-E-Qasim.

A total of 30 independent soil samples were collected from three different localities in Karachi, Sindh, Pakistan (Sabir Sre, DHA; Aziz Bhatti Public Park; and Bagh Ibn-E-Qasim) in October 2023 (Figure 1). The sites were chosen for their practical accessibility, ecological diversity, and informal field observations. At each site, 10 independent soil samples were taken, each weighing approximately 1 kg. To ensure the reliability and consistency of the data, each of these samples was composed of three subsamples taken at a depth of 10–25 cm using a garden shovel. The three subsamples were combined and treated as a single independent sample. The soil samples were labeled, placed in polyethylene bags to prevent moisture loss, and transported to the laboratory under cool conditions. Before isolating EPNs, the samples were sieved to remove stones, organic matter, and debris.

### 2.2. Isolation of Entomopathogenic Nematodes

The isolation of EPNs from the soil samples was performed using the insect baiting technique described by Bedding and Akhurst [10]. Each soil sample was divided into two portions and placed in plastic jars (500 mL, 12 cm in diameter). Four *Galleria mellonella* larvae were introduced onto the soil surface in each jar. The jars were incubated in darkness at 23 ± 1 °C, and they were inverted daily to ensure even moisture distribution. Water was added as needed to maintain optimal soil moisture during the baiting period. After a 7-day incubation, the *G. mellonella* larvae were removed, and if no EPNs were detected, the baiting process was repeated. Larvae showing signs of nematode infection were transferred to modified White traps [11] and maintained at 23 ± 1 °C. Infective juveniles (IJs) were collected from these traps and subjected to several cycles of exposure to fresh *G. mellonella* larvae to establish cultures for further study. After establishing cultures, approximately 20 IJs were morphologically identified according to the methods outlined by Nguyen 2007 [12] and subsequently measured under a compound microscope.

### 2.3. DNA Extraction and Sequencing

For molecular analyses, previously published protocols were followed, with some adjustments from Adams et al. 2007 [13]. Approximately 10 nematodes preserved in ethanol were used for DNA extraction. The nematodes were first rinsed with sterile water and transferred into a 0.2 mL microcentrifuge PCR tube, where they were mixed with 18 μL of 10 mM Tris, 1 mM EDTA, 1 μL of 2% Triton X, and 1 μL of Proteinase K (20 mg/mL, New England Biolabs, Ipswich, MA, USA). The nematode cuticles were disrupted by subjecting the sample to three freeze–thaw cycles using liquid nitrogen, followed by overnight incubation at −20 °C. The next day, the lysate was incubated at 56 °C for 1 h, then heated to 95 °C for 10 min. The PCR reaction was conducted with a total volume of 25 μL, containing 2 μL of genomic DNA as the template, 12.5 μL of 2X Taq Red Master Mix (Genesee Scientific, El Cajon, CA, USA), 1.25 μL each of forward and reverse primers (10 µM concentration), and 8 μL of nuclease-free water. Ribosomal DNA (rDNA) regions were first amplified to identify the nematode. For ITS rDNA, PCR amplification was performed using the primers TW81 (5′-GTTTCCGTAGGTGAACCTGC-3′) and AB28 (5′-ATATGCTTAAGTTCAGCGGGT-3′) [14] and the 28S rDNA gene was amplified using primers D2F (5′-CCTTAGTAACGGCGAGTGAAA-3′) and 536 (5′-CAGCTATCCTGAGGAAAC-3′) [15]. The same PCR conditions were used for amplifying all three loci: an initial denaturation step at 96 °C for 5 min, followed by 35 cycles of denaturation at 96 °C for 1 min, annealing at 57 °C for 45 s, and extension at 72 °C for 1 min, with a final extension at 72 °C for 10 min.

The PCR products were analyzed by electrophoresis on a 1% agarose gel stained with 0.0003% ethidium bromide, using a 1 kb plus DNA ladder (New England Biolabs) as a size reference. The products were then purified with the QIAquick^®^ PCR Purification Kit (Qiagen, Germantown, MD, USA). After purification, the samples were sequenced in both forward and reverse directions via Sanger sequencing at the UCR Core Instrumentation Facility. Any inconsistencies in the chromatogram sequences for each locus were reviewed and assembled using SeqManII software version 5.05 (DNASTAR, Madison, WI, USA). The consensus sequences were then compared with those in the National Center for Biotechnology Information (NCBI) GenBank database using the Basic Local Alignment Search Tool (BLAST). The sequences generated in this study have been deposited in NCBI GenBank.

### 2.4. Phylogenetic Analysis

To construct the heterhabditid phylogeny, ITS sequences from *Heterorhabditis* species and closely related taxa identified through BLAST were aligned using Clustal W in MEGA 11 with default settings. Manual adjustments were made to resolve alignment inconsistencies [16]. The program IQ-TREE [17] was utilized to select the optimal model for the ITS dataset using the “find best model” function and to generate a maximum-likelihood (ML) phylogenetic tree with ultrafast bootstrap support based on 1000 replicates.

For the steinernematid nematode phylogeny, ITS sequences obtained in this study were analyzed using BLAST at NCBI to identify closely related species. These sequences were aligned following the previously described approach. Similarly, 28S gene sequences of related species were identified and aligned. The ITS and 28S alignments for the same species were concatenated, and a phylogenetic tree was constructed using IQ-TREE, using a similar process as described above. 

### 2.5. Reproductive Potential

The reproductive potential of five strains of *Steinernema pakistanense* (Pak.S.SA.58; Pak.S.SA.62; Pak.S.SA.52; Pak.S.SA.63; Pak.S.SA.22) and three *Heterorhabditis indica* (Pak. H. BN.1; Pak.H.BN.3; Pak.H.BN.5) isolates was evaluated using a standardized bioassay. The assessment involved inoculating *G. mellonella* larvae with the nematodes to quantify the number of infective juveniles (IJs) produced. Fresh 100 IJs of each isolate were used, and the larvae were infected under controlled laboratory conditions. The emergence of IJs was monitored daily, and IJs were collected over a period of 10 days. The total number of IJs produced per larva was determined by counting IJs in three 1 mL subsamples. The reproductive potential was expressed as the mean number of IJs produced per infected *G. mellonella* larva for each *H. indica* and *S. pakistanense* isolate per day. Each isolate’s performance was measured in five multiple replicates to ensure reliability.

### 2.6. Rearing of Leucinodes Orbonalis

Infested eggplant/brinjal fruits (*Solanum melongena* L.) were collected from a local vegetable market and sliced to isolate different larval stages of the brinjal shoot and fruit borer (*L. orbonalis*). Fresh, uninfected brinjal fruits were disinfected and placed in sterile rearing containers. The larvae were introduced into small incisions made in the fruits to mimic natural infestation. The containers were kept at 25 ± 1 °C, with a 12:12 light photoperiod and 70% to 80% relative humidity [18].

### 2.7. Laboratory Experiment

Glass Petri dishes, 9 cm in diameter, were utilized in the bioassay to assess the efficacy of five strains of *Steinernema pakistanense* (Pak.S.SA.58; Pak.S.SA.62; Pak.S.SA.52; Pak.S.SA.63; Pak.S.SA.22) and three *Heterorhabditis indica* (Pak. H. BN.1; Pak.H.BN.3; Pak.H.BN.5) isolates against the brinjal shoot and fruit borer (*L. orbonalis*). Each dish was lined with Whatman No. 1 filter paper to maintain moisture. Infective juveniles (IJs) were applied at concentrations of 50, 150, and 200 IJs/mL prepared by first harvesting IJs from White traps and suspending them in sterile distilled water. The IJs were counted using a counting chamber under a compound microscope. A known volume of the nematode suspension was pipetted into the counting chamber, and the total number of IJs present was recorded. The initial concentration was adjusted by serial dilution with sterile distilled water to achieve the desired densities. For each experimental treatment, the calculated volume of the stock suspension was diluted in sterile distilled water to obtain final working concentrations of 50, 150, and 200 IJs/mL.

The control group received 1 mL of sterile distilled water without nematodes as a negative control. To prevent contamination between treatments, pipette tips were changed after each application. For each treatment, five mature larvae of *L. orbonalis* were individually placed in the Petri dishes. Each experiment was repeated three times with fresh insect batches and new nematode suspensions for each trial. The dishes were maintained at standard laboratory conditions, and observations were made 48 h post-inoculation. Mortality was recorded, and dead larvae were dissected under a stereomicroscope or placed in White traps to verify nematode infection through the emergence of IJs, following the method of White (1927) [19].

### 2.8. Microplot Experiment

A local variety of long eggplant/brinjal fruit (*Solanum melongena* L.) was cultivated during the brinjal growing season at the experimental field of the National Nematological Research Center in Karachi, Pakistan. The field, which had a known incidence of *Leucinodes orbonalis* infestation, was divided into three independent microplots, each measuring 1 m^2^ (1 m × 1 m) and consisting of two rows. To ensure replication in space, each nematode treatment and the untreated control were applied to multiple microplots, with three replicates per treatment group. This design accounted for spatial variability and minimized the risk of confounding factors, such as local environmental differences within the field. Microplots were monitored weekly for 5–8 weeks post-treatment, with observations focusing on signs of infestation, such as larvae, frass, and leaf damage, at a density of approximately 4 to 5 borers per plant.

The experimental design included three replicates for each of the three nematode treatments, Pak.S.SA.22, Pak.H.BN.3, and Pak.S.SA.52, alongside an untreated control group. Treatments were randomly allocated to the microplots to reduce potential bias. The nematode strains selected for this study, representing the *Steinernema* and *Heterorhabditis* genera, were chosen based on their high laboratory efficacy and minimal mortality rates. The control plots received an equivalent volume of sterile distilled water without nematodes to serve as a negative control. Each treatment involved the preparation of a liquid suspension of infective juveniles (IJs) in 1% glycerin, which served as a preservative to maintain the viability of the IJs during application. The suspension was prepared by dissolving the required number of IJs in water mixed with 1% glycerin, ensuring an even distribution of IJs within the solution. A dose of 2500 IJs per plant was applied to each plant in the microplots. As each microplot contained two plants, a total of 5000 IJs were applied per microplot (2500 IJs per plant × 2 plants). To convert the dose for field-scale applications, the dose was calculated based on the area of 1 m^2^ per microplot, with 10,000 microplots per hectare. This resulted in a dose of 50 million IJs per hectare. Nematodes were applied in the early morning, either at planting or upon detection of pest infestation, to ensure uniform distribution around the root zone. Nylon nets were erected around each microplot to maintain experimental integrity.

Applications of the nematode suspension were conducted weekly, with the initial application occurring one week after detecting pest infestation, followed by a second application one week later, and a third application one week after the second. Control plots received the same volume of water without IJs. Mortality was assessed two days after each application.

Dead larvae were then transported to the laboratory, where they were placed on White traps to confirm juvenile nematode emergence. This was recorded for each replicate in a completely randomized design.

### 2.9. Meteorological Data for the Experimental Area

A survey conducted in October 2023 recorded maximum daytime temperatures between 31 and 35 °C (average 33 °C). Soil temperatures at a depth of 5 cm were measured at 29.4 °C in the southern survey area and 31.2 °C in the eastern survey area. Minimum temperatures ranged from 24 to 29 °C (average 26.5 °C). Relative humidity decreased from 84% at the beginning of the month to 37% by the end, with total precipitation recorded at 1.4 mm. A microplot experiment was conducted at the National Nematological Research Centre, University of Karachi, from September 2024 to October 2024. In September, the average maximum temperature rose to 33 °C, with the average low remaining at 27 °C. Precipitation in September totaled 42 mm, while the relative humidity averaged 75%. In October 2024, the maximum daytime temperature ranged from 33 °C to 37 °C (average 35 °C), and the minimum temperature varied between 24 °C and 27 °C (average 25.5 °C). The average relative humidity for October was 65%, and the total precipitation recorded throughout the month was 12.9 mm.

### 2.10. Statistical Methodology

All statistical analyses were conducted using SAS (version 9.1, SAS Institute, Cary, NC, USA), and results were expressed as mean ± standard error (SE), with significance set at *p* < 0.05. Data were corrected using Abbott’s formula to account for natural mortality in the control group:

$$Correct\;Mortality\;\%=\left(\frac{Observed\;Mortality-Control\;Mortality}{100-Control\;Mortality}\right)\times 100$$

The Shapiro–Wilk test was conducted to assess normality before performing a one-way ANOVA to evaluate significant differences among treatment groups using the model:

$$Y_{ij}=\mu +\tau_{i}+\varepsilon_{ij}$$

*Y_ij_* = observed response (e.g., mortality rate); µ = overall mean; T_i_ = effect of the treatment; *ε_ij_* = random error.

To identify significant differences between treatment means, Duncan’s Multiple Range Test (DMRT) was performed:

$$Q=\frac{\left|M_{i}-M_{j}\right|}{\sqrt{MSW/n}}$$

The median lethal dose (LD_50_) was estimated using Probit Analysis, modeled as:

Y=a+bX

*Y* = Probit-transformed mortality; *a* = intercept; *b* = slope; *X* = log-transformed time.

For field experiments, repeated measures ANOVA was conducted to test the effect of multiple spray applications over time. Pearson’s correlation coefficient was used to examine the relationship between nematode reproductive potential and pest mortality:

$$r=\frac{\sum \left(X_{i}-\bar{X})(Y_{i}-\bar{Y}\right)}{\sqrt{\sum {\left(X_{i}-\bar{X}\right)}^{2}\sum {\left(Y_{i}-\bar{Y}\right)}^{2}}}$$

*X_i_*, *Y_i_* = individual values for nematode reproduction and mortality; *X*, *Y* = respective means.

## 3. Results

### 3.1. Field Surveys

The analysis of EPN distribution across sampled localities revealed that *Heterorhabditis indica* and *Steinernema pakistanense* were the predominant species identified. *Zoysia japonica* was the most frequently recorded host plant, with four occurrences at Sabir Sre, DHA, indicating favorable conditions for EPN proliferation in this locality. In contrast, Aziz Bhatti Public Park exhibited a greater diversity of EPNs, including those associated with *Pongemia pinnata*, *Conocarpus erectus*, *Cassia fistula*, and *Ficus carica*. This suggests that Aziz Bhatti Public Park provides varied habitat suitability, supporting a broader range of EPN species (Table 1). Morphologically, the measurements of infective juveniles (IJs) from Pak.S.SA.58, Pak.S.SA.62, Pak.S.SA.52, Pak.S.SA.63, and Pak.S.SA.22 fall within the range of *S. pakistanense*, while three species, Pak. H. BN.1, Pak.H.BN.3, and Pak.H.BN.5, belong to *H. indica*.

### 3.2. Molecular Identification and Phylogenic Analysis

Amplification of ribosomal DNA from *Heterorhabditis* nematodes using the TW81/AB28 primers generated fragments ranging from 661 bp to 770 bp. Comparison with available NCBI sequences revealed 99% identity to the KY977412 sequence of *Heterorhabditis indica* isolates Pak.H.BN.1, Pak.H.BN.2, and Pak.H.BN.3, confirming that all isolates belonged to *H. indica*. Similarly, the ITS sequences of five *Steinernema* isolates ranged in length from 441 bp to 803 bp. Comparison with NCBI database sequences indicated a similarity of approximately 99–100% to *Steinernema pakistanense* isolates, associated with accession numbers JX135548, AY748449, JN157771, MK491798, and JX135546. Similarly, the 28S gene fragments of *Steinernema* isolates, ranging from 845 bp to 885 bp, showed over 99% similarity to *Steinernema pakistanense* isolates, with accession numbers PP334013 and KC625523. The accession numbers for the ITS sequences of *Steinernema* spps. are PQ562434, PQ562435, PQ562445, PQ562446, and PQ562447, while the D2–D3 rDNA sequences of *Steinernema* spp. are registered under PQ566940, PQ566941, PQ566942, PQ566943, and PQ566944. Additionally, the accession numbers for the three ITS sequences of *Heterorhabditis* spp. are PQ562327, PQ562352, and PQ562427.

**Figure 2:**
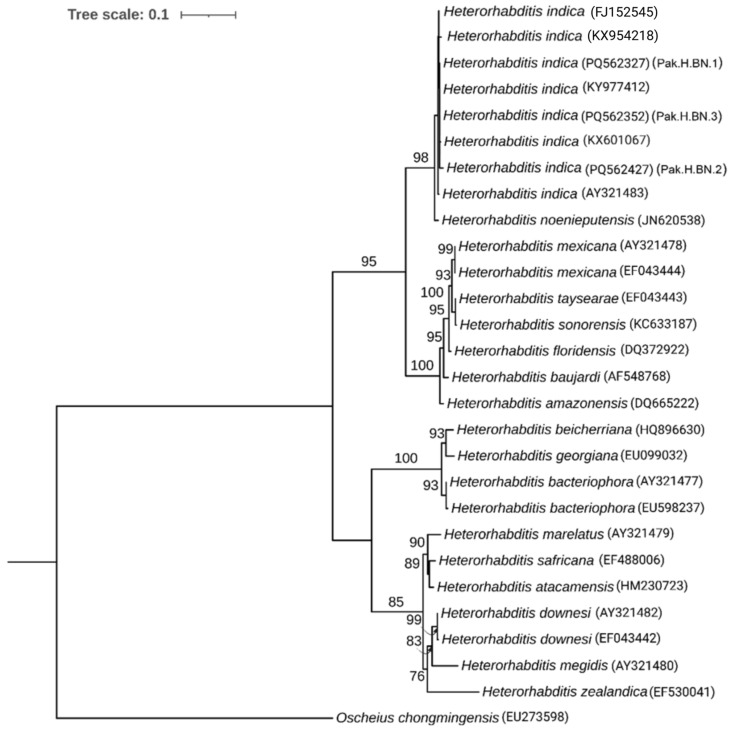
A maximum likelihood phylogenetic tree constructed using publicly available *Heterorhabditis* species sequences and newly obtained sequences based on the ITS gene. The analysis was performed with 1000 bootstrap replicates, and the numbers on the branches indicate bootstrap support values for each node. The sequences for *H. indica* (FJ152545; *H. gerrardi*) and *H. indica* (KX954218; *H. pakistanensis*) are ascribed to species names that are junior synonyms of *H. indica* [20].

For the phylogenetic analysis of *Heterorhabditis*, three sequences from this study, along with 24 sequences representing various *Heterorhabditis* species identified through BLAST, were included. The ITS sequence of *Oscheius chongmingensis* (accession number EU273598) was used as an outgroup. Using the IQ-tree program, the “TIM2+F+I” model was identified as the best-fit model based on the Bayesian Information Criterion. The phylogenetic analysis revealed that all isolates from this study clustered together with *Heterorhabditis indica*, positioned adjacent to *Heterorhabditis noenieputenis* (Figure 2).

For the phylogenetic analysis of steinernematids, five ITS and five 28S gene sequences obtained in this study, along with 45 sequences representing various *Steinernema* species identified via BLAST, were used to construct a concatenated phylogenetic tree. All steinernematids and their corresponding GenBank accession numbers included in the phylogenetic analysis are listed in Table 2.

**Figure 3:**
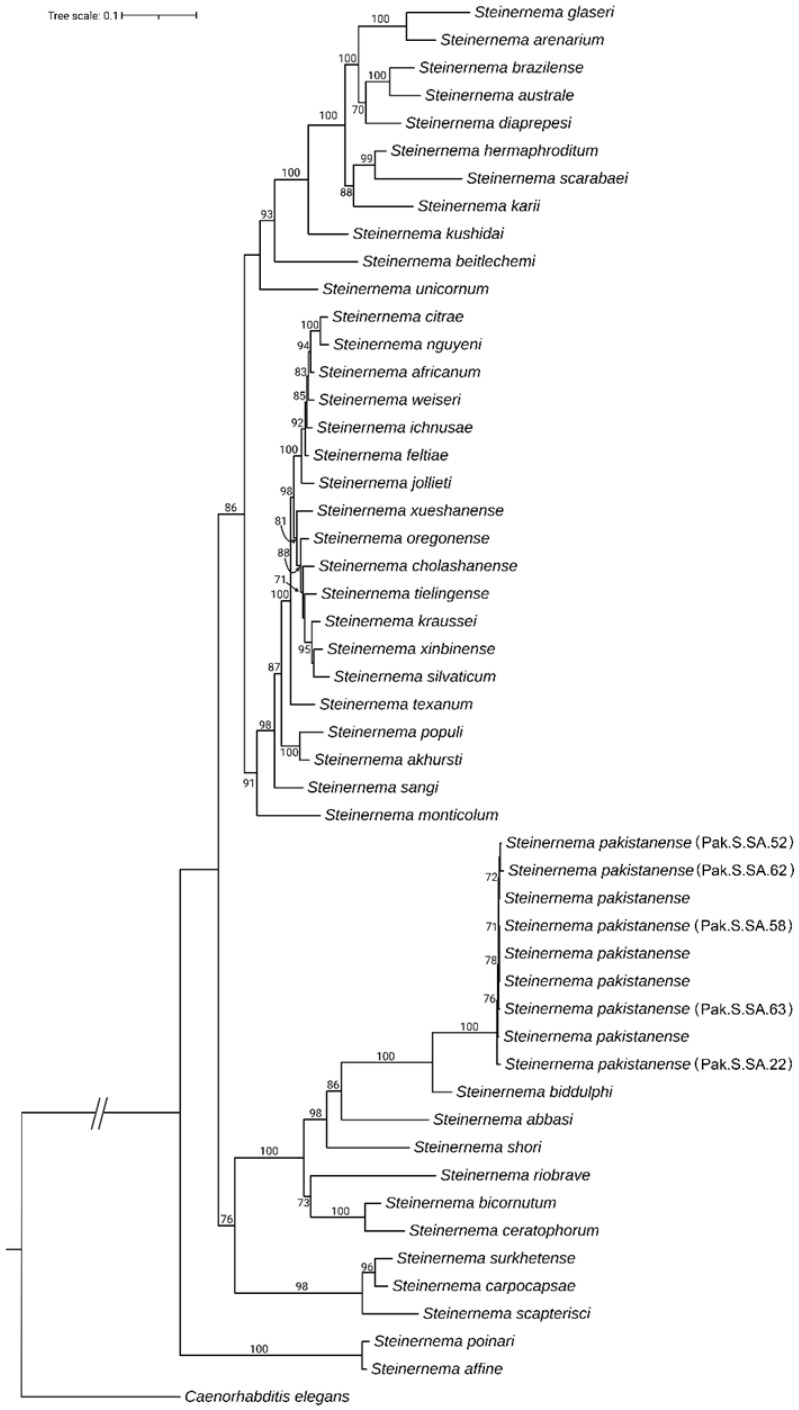
The maximum likelihood phylogenetic relationships among various *Steinernema* species were inferred using ITS and D2–D3 regions of 28S rDNA gene sequences, analyzed with the GTR+F+G4 model as determined by the Bayesian Information Criterion. The analysis included 1000 bootstrap replicates, with branch numbers representing bootstrap support values for each node.

The concatenated ITS and 28S sequence of *Caenorhabditis elegans* (accession numbers X03680 and MW646314) were included as outgroups. The IQ-tree program identified the “GTR+F+G4” model as the best fit based on the Bayesian Information Criterion. The analysis showed that all *Steinernema pakistanense* isolates from this study clustered with previously described *S. pakistanense* and were positioned next to *Steinernema biddulphi* (Figure 3).

### 3.3. Reproductive Potential

**Figure 4:**
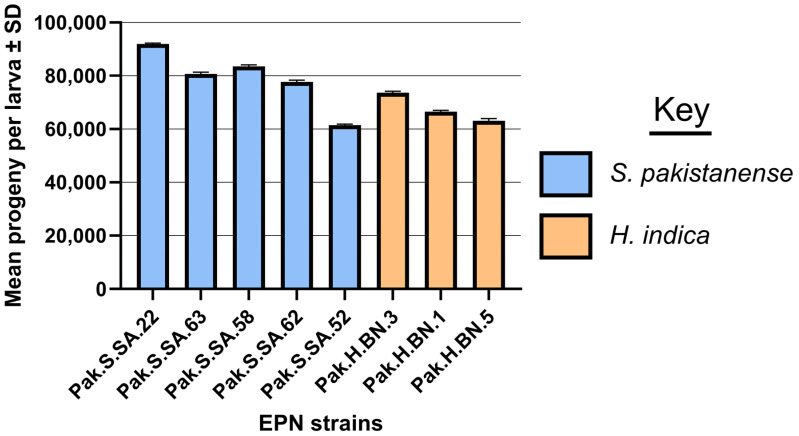
Reproductive potential of eight strains of entomopathogenic nematodes in *Galleria mellonella* larvae.

The reproductive potential of eight entomopathogenic nematode (EPN) strains was evaluated, and the results indicated significant differences among the strains (Figure 4). The mean number of progeny per larva varied across the strains, with Pak.S.SA.22 exhibiting the highest reproductive potential, producing an average of 91,944 progeny per larva (±366). This was followed by Pak.S.SA.63 (80,743 ± 653) and Pak.S.SA.58 (83,533 ± 577). The strain Pak.S.SA.62 produced 77,693 progeny per larva (±671), while Pak.H.BN.3, Pak.H.BN.1, and Pak.H.BN.5 produced (73,600 ± 529), (66,533 ± 472), and (63,130 ± 704) progeny per larva, respectively. The lowest reproductive potential was observed in Pak.S.SA.52, which produced 61,453 progeny per larva (±410). Statistical analysis using one-way ANOVA revealed a highly significant difference among the strains (F = 95.08; *p* < 0.001). These results indicate that strains Pak.S.SA.22, Pak.S.SA.63, and Pak.S.SA.58 significantly outperformed the other strains in terms of reproductive potential, suggesting that they may have greater efficacy as biocontrol agents against targeted pest larvae.

### 3.4. Laboratory Experiment

**Figure 5:**
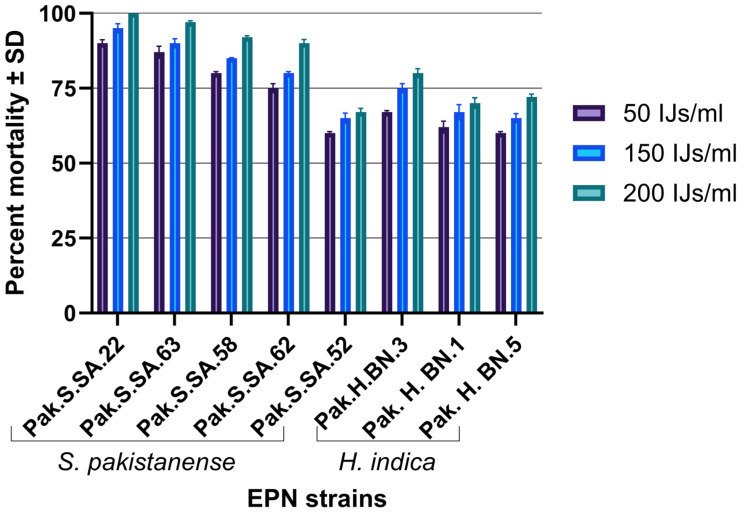
Laboratory evaluation of *Leucinodes orbonalis* mortality treated with eight strains of entomopathogenic nematode.

The effectiveness of different concentrations of infective juveniles (IJs) was evaluated on various strains of the host. The treatment groups consisted of 50 IJs/mL, 150 IJs/mL, and 200 IJs/mL. The results indicated varying levels of effectiveness across the strains tested, with Pak.S.SA.22 achieving the highest efficacy at 100% for the 200 IJs/mL treatments, while Pak.H.BN.5 exhibited the lowest efficacy with 72% at the same concentration (Figure 5). A one-way ANOVA was conducted to compare the means among the three groups. The summary statistics revealed an average efficacy of 72.63% for the 50 IJs/mL group, 77.75% for the 150 IJs/mL group, and 83.50% for the 200 IJs/mL group. Mortality was not observed in the control treatment, which was the addition of 1 ml of water without nematodes. The analysis yielded a significant F-value of 1.58 with a corresponding *p*-value of 0.230, indicating no statistically significant difference in mean efficacy among the treatment groups (F crit = 3.467). The comparison of variance between groups (SS = 473.58) and within groups (SS = 3153.38) further supports the lack of significant treatment effects. The LD 50 values of Pak.S.SA.22, Pak.S.SA.63, Pak.S.SA.58, Pak.S.SA.62, Pak.H.BN.3, Pak.H.BN.1, Pak.H.BN.5, and Pak.S.SA.52 were calculated as 14.38, 7.59, 11.71, 19.43, 32.37, 53.85, 71.65, and 79.33 IJs/mL, respectively.

### 3.5. Microplot Experiment

**Figure 6:**
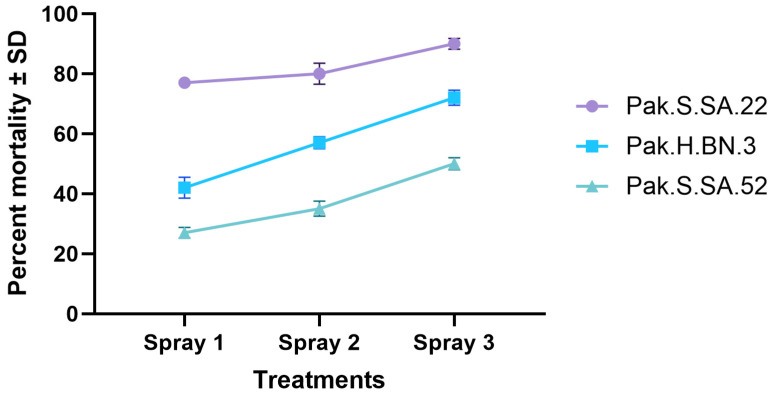
*Leucinodes orbonais* mean mortality treated with three different spray events of entomopathogenic nematodes in microplot trial.

The effectiveness of the three tested EPN strains (Pak.S.SA.22, Pak.H.BN.3, and Pak.S.SA.52) varied significantly across the three spray applications. Statistical analysis revealed notable differences in pest reduction percentages, indicating the varying biocontrol potential of the strains under field conditions. The negative control, water only, exhibited no effect against the pest. The strain Pak.S.SA.22 exhibited the highest pest suppression efficacy, with mean reductions of 77%, 80%, and 90% after the first, second, and third sprays, respectively. This progressive and statistically significant increase in efficacy (*p* < 0.01) suggests improved nematode establishment and activity with repeated applications. The strain Pak.H.BN.3 achieved moderate pest suppression, with reductions of 42%, 57%, and 72%, respectively, across the three sprays. Although less effective than Pak.S.SA.22, it displayed consistent improvement, making it a viable option for supplemental pest management. In contrast, Pak.S.SA.52 recorded the lowest reduction percentages, starting at 27% and increasing to 35% and 50% after the second and third sprays (Figure 6). Despite its lower efficacy, Pak.S.SA.52 may still contribute to integrated pest management (IPM) strategies when used in combination with other control measures. The repeated measures ANOVA confirmed a significant main effect of spray applications on pest reduction (F = 18.79, *p* = 0.0093), while Tukey’s post-hoc test demonstrated that the third spray was significantly more effective than the first and second sprays (*p* < 0.05). Correlation analysis revealed significant positive relationships among the efficacies of the three strains, with Pak.S.SA.22 and Pak.H.BN.3 showing a correlation of r = 0.63, *p* < 0.05, Pak.H.BN.3 and Pak.S.SA.52 exhibiting r = 0.81, *p* < 0.05, and Pak.S.SA.22 and Pak.S.SA.52 displaying r = 0.74, *p* < 0.05. These findings indicate the potential complementary effects of the strains. The progressive increase in pest suppression observed for all three strains suggests a dose-dependent or cumulative effect, likely due to improved nematode establishment, enhanced infection rates, or successive reduction in the pest population over time. The superior performance of Pak.S.SA.22 underscores its potential as a primary biocontrol agent for *L. orbonalis*, while the moderate performance of Pak.H.BN.3 and Pak.S.SA.52 suggests their utility as secondary options or in combination with Pak.S.SA.22.

## 4. Discussion

The increasing reliance on chemical pesticides for managing agricultural pests, particularly the brinjal fruit and shoot borer *Leucinodes orbonalis*, highlights the need for sustainable pest control methods [6]. This study explores the efficacy of indigenous entomopathogenic nematodes (EPNs), specifically isolates of *Steinernema pakistanense* and *Heterorhabditis indica*, as promising biological control agents against *L. orbonalis* in Pakistan. The effectiveness of EPNs as biocontrol agents is influenced significantly by the specific strains used. Even within the same species, different strains exhibit variations in virulence, adaptability, and reproductive potential, all of which are critical factors for pest suppression. Strain selection is thus vital for achieving optimal results, as locally adapted strains are often more effective against target pests and environmental conditions [21,22]. The isolation of EPNs from local soil samples revealed that *H. indica* and *S. pakistanense* were predominant in the surveyed areas, indicating their ecological adaptability to local conditions. Regular surveys conducted across diverse habitats in Pakistan have led to the identification of fifteen species of EPNs, including twelve species of *Steinernema* and three species of *Heterorhabditis* [23]. Molecular identification using ITS and 28S sequences confirmed that all *Heterorhabditis* isolates belonged to *H. indica*, while the *Steinernema* isolates were identified as *S. pakistanense*. High sequence similarity (99–100%) with NCBI reference sequences and clustering patterns in phylogenetic analyses further validated these classifications. Morphological measurements of infective juveniles (IJs) aligned with the described ranges for *S. pakistanense* and *H. indica*, providing additional confirmation of their identity.

Our findings that Pak.S.SA.22 exhibited the highest reproductive potential (91,944 progeny per larva) align with previous research, indicating a higher nematode density often correlates with enhanced lethality against host insects [24]. The significant variation in the infective capabilities of the EPN strains observed in this study is consistent with earlier studies, which reported differences in virulence among various EPN species [22]. Pak.H.BN.5 showed a lower efficacy (72%) compared to Pak.S.SA.22, which achieved 100% efficacy at the highest concentration of 200 IJs/mL. This discrepancy in performance could be attributed to factors such as the nematode’s ability to locate and penetrate the larvae and the particular pathogenicity of the strains involved [9].

Previous studies have documented the effectiveness of EPNs in controlling various pests, affirming the importance of local strains in enhancing pest management outcomes [25,26]. The application of higher concentrations of IJs resulted in improved efficacy, which supports the notion that initial pest populations significantly influence the success of biocontrol interventions [27]. The LD50 values calculated for the various EPN strains underscore their relative potencies and can guide future application protocols. The calculated LD50 values for the strains ranged from 7.59 to 79.33 IJs/mL, positioning Pak.S.SA.63 and Pak.S.SA.22 as particularly effective candidates for further exploration. These values indicate that optimizing nematode concentrations in field applications is crucial to maximize pest control while minimizing economic impacts. The findings of our study align with previous research emphasizing the potential of Indigenous EPN strains against *Leucinodes orbonalis*. For instance, Muhammad et al. 2024 [28] demonstrated substantial mortality rates of *L. orbonalis* larvae following exposure to local EPN strains, reinforcing our findings regarding the efficacy of *S. pakistanense* strains. Similar conclusions were drawn by Hussaini et al. 2002 [29], who reported significant larval mortality when specific local EPN strains were applied against *L. orbonalis*. Limited research has been conducted on the use of EPNs against the brinjal fruit and shoot borer despite it being a serious pest. Further studies are needed to explore the potential of EPNs as effective biocontrol agents for its management. Ganga et al. achieved notable success in employing EPN applications to manage *L. orbonalis* during field trials conducted over three consecutive years, emphasizing the significance of research in promoting sustainable alternatives to chemical insecticides [24]. The results from the microplot experiments demonstrated significant reductions in pest populations, particularly with Pak.S.SA.22, which showed a progressive increase in efficacy across multiple applications. Different studies have reinforced the benefits of repeated applications of EPNs in pest management [8,30,31]. The moderate performance of Pak.H.BN.3 and the lower effectiveness of Pak.S.SA.52 suggest the importance of conducting multi-strain evaluations to identify the most effective combinations for field applications, which can optimize pest suppression while minimizing chemical pesticide use.

The economic and ecological implications of utilizing EPNs are significant. Economically, EPNs can reduce crop losses by effectively controlling pest populations, potentially lowering the costs associated with chemical pesticide applications. Ecologically, EPNs are considered safe for non-target organisms, including plants and animals, making them suitable candidates for integrated pest management and sustainable agriculture [32]. Our study’s findings are particularly relevant in the context of increasing concerns over pesticide use and resistance development in agricultural settings. Excessive reliance on chemical insecticides for managing pests like *L. orbonalis* has detrimental consequences, including impacts on human health, non-target organisms, and the broader ecosystem. These factors reinforce the need for sustainable alternatives such as EPNs, which are regarded as eco-friendly and safer for beneficial organisms [33].

## 5. Conclusions

In conclusion, the findings from this study provide compelling evidence for the efficacy of Indigenous EPN strains as biocontrol agents against the brinjal fruit and shoot borer *Leucinodes orbonalis*. By demonstrating the potential of *S. pakistanense* and *H. indica* strains to safely manage this significant pest, the research supports the development of integrated pest management strategies that reduce dependency on chemical insecticides.

## Figures and Tables

**Table 1:** Description of the various locations of different entomopathogenic nematodes isolated from the field along with associated vegetation in Karachi, Pakistan.

Species Code	Vegetation	Location	GPS Coordinates	IJ Length (µm)
Pak. H. BN.1	*Zoysia japonica*	Sabir Sre, DHA	24.8388°	528
			67.0439°	(479–573)
Pak.S.SA.58	*Pongemia pinnata*	Aziz Public Bhatti Park	24.9152°	662
			67.0948°	(649–716)
Pak.H.BN.3	*Zoysia japonica*	Sabir Sre, DHA	24.8379°	511
			67.0441°	(479–573)
Pak.S.SA.62	*Conocarpus erectus*	Aziz Bhatti Public Park	24.9153°	678
			67.0952°	(649–716)
Pak.H.BN.2	*Zoysia japonica*	Sabir Sre, DHA	24.8378°	525
			67.0444°	(479–573)
Pak.S.SA.52	*Cassia fistula*	Aziz Bhatti Public	24.9139°	663
			67.0965°	(649–716)
Pak.S.SA.63	*Ficus carica*	Aziz Bhatti Public Park	24.9134°	681
			67.0953°	(649–716)
Pak.S.SA.22	*Pongemia pinnata*	BAGH Ibn-E-Qasim	24.8078°	669
			67.0225°	(649–716)

**Table 2:** Information on various *Steinernema* species, including their geographical locations and GenBank accession numbers, used to construct the concatenated phylogenetic tree.

Nematode Species	Geographic Origin (28S)	Geographic Origin (ITS)	GenBank Accession No.	
			28S	ITS
*S. abbasi*	-	Pakistan	MN533913	EF469773
*S. affine*	UK	Turkey	AF331899	PP538017
*S. africanum*	Rwanda	Rwanda	OM423154	ON041032
*S. akhursti*	China	China	GU395638	DQ375757
*S. arenarium*	Slovakia	Russia	KU194619	KU194614
*S. australe*	Chile	Chile	FJ235126	FJ235125
*S. beitlechemi*	South Africa	South Africa	KT580949	KT373856
*S. bicornutum*	Serbia	Yugoslavia	GU569045	AF121048
*S. biddulphi*	South Africa	South Africa	KT580950	KT373857
*S. brazilense*	Brazil	Brazil	FJ410326	FJ410325
*S. carpocapsae*	Colombia	Bulgaria	MK558056	AF121049
*S. ceratophorum*	-	China	MW029452	AY230165
*S. cholashanense*	China	India	EF520284	MH065747
*S. citrae*	South Africa	South Africa	MF540676	FJ235074
*S. diaprepesi*	USA	USA	GU177828	AF122021
*S. feltiae*	USA	USA	AF331906	AF121050
*S. glaseri*	USA	USA	GU177831	AF122015
*S. hermaphroditum*	India	India	MF693228	MF663703
*S. ichnusae*	Sardinia	Sardinia	EU421130	EU421129
*S. jollieti*	USA	USA	GU569051	AY171265
*S. karii*	-	Kenya	AF331902	AY230173
*S. kraussei*	Poland	Germany	KC631424	AY230175
*S. kushidai*	Japan	China	AF331897	GQ497741
*S. monticolum*	-	Korea	GU395647	AF122017
*S. nguyeni*	South Africa	South Africa	KR815816	KP325084
*S. oregonense*	USA	USA	GU569055	AF122019
*S. pakistanense*	India	India	MF289982	MK491798
*S. pakistanense*	India	Pakistan	MF289983	JN157771
*S. pakistanense*	-	-	KC625523	GQ497273
*S. pakistanense*	India	Pakistan	PP334013	JX135548
*S. pakistanense* Pak.S.SA.58	Pakistan *	Pakistan *	PQ566940	PQ562434
*S. pakistanense* Pak.S. SA.62	Pakistan *	Pakistan *	PQ566941	PQ562435
*S. pakistanense* Pak.S. SA.52	Pakistan *	Pakistan *	PQ566942	PQ562445
*S. pakistanense* Pak.S. SA.63	Pakistan *	Pakistan *	PQ566943	PQ562446
*S. pakistanense* Pak.S. SA.22	Pakistan *	Pakistan *	PQ566944	PQ562447
*S. poinari*	Czech Republic	Czech Republic	KF241750	KF241753
*S. populi*	China	China	MZ367685	MZ367621
*S. riobrave*	USA	USA	GU177834	GU174000
*S. sangi*	India	Vietnam	MF620997	AY355441
*S. scapterisci*	-	Uruguay	GU395646	AF122020
*S. scarabaei*	USA	Chile	AY172023	FJ263673
*S. shori*	India	India	OR194555	OR194554
*S. silvaticum*	Poland	Poland	KC631426	MG543848
*S. surkhetense*	India	India	KU187262	MF919614
*S. texanum*	USA	USA	EF152569	EF152568
*S. tielingense*	China	China	GU994202	GU994201
*S. unicornum*	Chile	Chile	GU191462	GQ497167
*S. weiseri*	France	Czech Republic	FJ165549	KJ696685
*S. xinbinense*	China	China	GU994204	JN171593
*S. xueshanense*	China	China	FJ666053	FJ666052
*Caenorhabditis elegans*	-	-	X03680	MW646314

Note: - information not available; * sequenced during this study.

## Data Availability

The sequence data generated during this study are available in the NCBI database under the following accession numbers: PQ562434, PQ562435, PQ562445, PQ562446, PQ562447, PQ566940, PQ566941, PQ566942, PQ566943, PQ566944, PQ562327, PQ562352, and PQ562427. Additional data from this study are available upon request from the corresponding authors.

## References

[1] Plazas M., Prohens J., Cuñat A.N., Vilanova S., Gramazio P., Herraiz F.J., Andújar I. (2014). Reducing Capacity, Chlorogenic Acid Content and Biological Activity in a Collection of Scarlet (*Solanum aethiopicum*) and Gboma (*S. macrocarpon*) Eggplants. Int. J. Mol. Sci..

[2] Taylo L., Sison M.L., Hautea D. (2016). Use of Artificial Infestation for Field Bioefficacy Assessment of Bt Eggplant against the Eggplant Fruit and Shoot Borer, *Leucinodes orbonalis* Guenee (Lepidoptera: Crambidae). Philipp. Agric. Sci..

[3] Dhaliwal G.S., Singh R., Chhillar B.S. (2016). Essentials of Agricultural Entomology.

[4] Javed H., Mukhtar T., Javed K., Moshin A. (2017). Management of Eggplant Shoot and Fruit Borer (*Leucinodes orbonalis* Guenee) by Integrating Different Non-Chemical Approaches. Pak. J. Agric. Sci..

[5] Khanal D., Pandey R., Dhakal R., Neupane N., Shrestha A., Nepali Joseph M., Paudel A., Pandey M. (2021). Efficacy of Bio-Rational Pesticides for the Management of *Leucinodes orbonalis* Guenee in Rupandehi, Nepal. Heliyon.

[6] Atreya K. (2008). Probabilistic Assessment of Acute Health Symptoms Related to Pesticide Use under Intensified Nepalese Agriculture. Int. J. Environ. Health Res..

[7] Lu D., Macchietto M., Chang D., Barros M.M., Baldwin J., Mortazavi A., Dillman A.R. (2017). Activated Entomopathogenic Nematode Infective Juveniles Release Lethal Venom Proteins. PLoS Pathog..

[8] Mengal M.A., Javed S., Majeed S. (2024). Efficacy of Entomopathogenic Nematodes in Laboratory and Field Conditions of *Cicer arietinum* against Cotton Bollworm, *Helicoverpa armigera* Hübner (Lepidoptera: Noctuidae). Egypt. J. Biol. Pest. Control.

[9] Fayyaz S., Javed S., Rumbos C.I., Athanassiou C.G., Abd-Elgawad M.M.M., Askary T.H., Coupland J. (2017). Control of Stored Grain Pests by Entomopathogenic Nematodes. Biocontrol Agents: Entomopathogenic and Slug Parasitic Nematodes.

[10] Bedding R., Akhurst R. (1975). A Simple Technique for the Detection of Insect Parasitic Rhabditid Nematodes in Soil. Nematologica.

[11] Kaya H.K., Patricia Stock S., Lacey L.A. (1997). Techniques in Insect Nematology. Manual of Techniques in Insect Pathology.

[12] Nguyen K.B., Nguyen K., Hunt D. (2007). Methodology, Morphology and Identification. Nematology Monograph and Perspectives: Entomopathogenic Nematodes: Systematics, Phylogeny and Bacterial Symbionts.

[13] Adams B.J., Peat S.M., Dillman A.R., Nguyen K., Hunt D. (2007). Phylogeny And Evolution. Entomopathogenic Nematodes: Systematics, Phylogeny and Bacterial Symbionts.

[14] Joyce S., Reid A., Driver F., Curran J., Burnell A.M., Ehlers R.-U., Masson J.P. (1994). Application of Polymerase Chain Reaction (PCR) Methods to the Identification of Entomopathogenic Nematodes. Genetics of Entomopathogenic Nematode-Bacterium Complexes. Proceedings and National Reports 1990–1993, St. Patrick’s College, Maynooth, Co. Kildare, Ireland.

[15] Nguyen K.B., Malan A.P., Gozel U. (2006). *Steinernema khoisanae* n. Sp. (Rhabditida: Steinernematidae), a New Entomopathogenic Nematode from South Africa. Nematology.

[16] Tamura K., Stecher G., Kumar S. (2021). MEGA11: Molecular Evolutionary Genetics Analysis Version 11. Mol. Biol. Evol..

[17] Nguyen L.-T., Schmidt H.A., Von Haeseler A., Minh B.Q. (2015). IQ-TREE: A Fast and Effective Stochastic Algorithm for Estimating Maximum-Likelihood Phylogenies. Mol. Biol. Evol..

[18] Sharma A., Solan N., Pradesh H., Rajinder Singh Rana I., Chander Sharma K., Singh S., Kumar A., Aarti Sharma C., Singh Rana R. (2017). Biology of Brinjal Shoot and Fruit Borer (*Leucinodes orbonalis* Guenee) on Brinjal Crop under Laboratory Conditions. J. Entomol. Zool. Stud..

[19] White G.F. (1927). A Method for Obtaining Infective Nematode Larvae from Cultures. Science.

[20] Hunt D.J., Subbotin S.A., Hunt D.J., Nguyen K.B. (2016). Taxonomy and Systematics. Advances in Entomopathogenic Nematode Taxonomy and Phylogeny.

[21] Grewal P.S., Ehlers R.U., Shapiro-Ilan D.I. (2005). Nematodes as Biocontrol Agents.

[22] Abd-Elgawad M.M.M., Abd-Elgawad M.M.M., Askary T.H., Coupland J. (2017). Status of Entomopathogenic Nematodes in Integrated Pest Management Strategies in Egypt. Biocontrol Agents: Entomopathogenic and Slug Parasitic Nematodes.

[23] Khan S., Javed S., Khanum T.A., Kazi N. (2021). Entomopathogenic Nematode (Nematoda: Rhabditida) Survey and Their Occurrence in Soil of District Lakki Marwat, Khyber Pakhtunkhwa Province, Pakistan. Punjab Uni. J. Zool..

[24] Ganga Visalakshy P.N., Krishnamoorthy A., Hussaini S.S. (2009). Field Efficacy of Entomopathogenic Nematode *Steinernema carpocapsae* (Weiser, 1955) against Brinjal Shoot and Fruit Borer, *Leucinodes orbonalis* Guenee. Nematol. Mediterr..

[25] Banu G., Gulsarbanu J., Subaharan K., Iyer R. (2004). Occurrence and Distribution of Entomopathogenic Nematodes in White Grub Endemic Areas. Occurrence and Distribution of Entomopathogenic Nematodes in White Grub Endemic Areas of Kerala. J. Plant Crops.

[26] Abd-Elgawad M.M.M., Spiridonov S.E. (2014). Entomopathogenic Nematode Application in Egypt and Russia: Challenges and Opportunities. Egypt. J. Agron..

[27] Patuwatha Withanage D.B.M., Briar S.S., Edeogu I. (2024). Efficacy of Commercially Available Entomopathogenic Nematodes against Insect Pests of Canola in Alberta, Canada. J. Helminthol..

[28] Ataullah M., Riaz S., Tariq M., Aslam A., Qadir A., Asghar A., Shehzad M., Akhtar M.F. (2024). In Vitro Exploration of Entomopathogenic Nematodes as Potential Biocontrol Agents of Brinjal Borer *Leucinodes orbonalis* Guenée (Lepidoptera: Crambidae). Plant Prot..

[29] Hussaini S.S., Singh S.P., Nagesh M. (2002). In Vitro and Field Evaluation of Some Indigenous Isolates of *Steinernema* and *Heterorhabditis indica* against Shoot and Fruit Borer, *Leucinodes orbonalis*. Indian J. Nematol..

[30] Lola-Luz T., Downes M., Dunne R. (2005). Control of Black Vine Weevil Larvae *Otiorhynchus sulcatus* (Fabricius) (Coleoptera:Curculionidae) in Grow Bags Outdoors with Nematodes. Agric. For. Entomol..

[31] Lola-Luz T., Downes M. (2007). Biological Control of Black Vine Weevil *Otiorhynchus sulcatus* in Ireland Using *Heterorhabditis megidis*. Biol. Control.

[32] Fallet P., Bazagwira D., Ruzzante L., Ingabire G., Levivier S., Bustos-Segura C., Kajuga J., Toepfer S., Turlings T.C.J. (2024). Entomopathogenic Nematodes as an Effective and Sustainable Alternative to Control the Fall Armyworm in Africa. Proc. Natl. Acad. Sci. USA Nexus.

[33] Shapiro-Ilan D.I., Leite L.G., Han R., Morales-Ramos J.A., Rojas M.G., Shapiro-Ilan D.I. (2023). Production of Entomopathogenic Nematodes. Mass Production of Beneficial Organisms: Invertebrates and Entomopathogens.

